# Could gut microbiota serve as prognostic biomarker associated with colorectal cancer patients' survival? A pilot study on relevant mechanism

**DOI:** 10.18632/oncotarget.10064

**Published:** 2016-06-15

**Authors:** Zhiliang Wei, Shougen Cao, Shanglong Liu, Zengwu Yao, Teng Sun, Yi Li, Jiante Li, Dongfeng Zhang, Yanbing Zhou

**Affiliations:** ^1^ Department of General Surgery, Affiliated Hospital of Qingdao University, Qingdao, China; ^2^ Department of General Surgery, Yantai Yuhuangding Hospital, Yantai, China; ^3^ Department of General Surgery, Qingdao Municipal Hospital Group, Qingdao, China; ^4^ Department of Epidemiology and Health Statistics, Qingdao University Medical College, Qingdao, China

**Keywords:** colorectal cancer, inflammation, intestinal microbiology, prognostic biomarker, prognosis

## Abstract

Evidences have shown that dysbiosis could promote the progression of colorectal cancer (CRC). However, the association of dysbiosis and prognosis of CRC is barely investigated. Therefore, we used 16S rRNA gene sequencing approach to determine differences in microbiota among tumor tissues of different prognosis and found that *Fusobacterium nucleatum* and *Bacteroides fragilis* were more abundant in worse prognosis groups, while *Faecalibacterium prausnitzii* displayed higher abundance in survival group. To further explore the prognostic value of the found bacteria, Kaplan–Meier and Cox proportional regression analyses were used and the results exhibited that high abundance of *F. nucleatum* and *B. fragilis* were independent indicators of poor patient's survival. Besides, the expression of major inflammatory mediator were analyzed using PCR and western blot methods, and it turned out that high abundance of *F. nucleatum* was associated with increased expression of TNF-α, β-catenin and NF-κB, while COX-2, MMP-9 and NF-κB were positively related with high *B. fragilis* level, and high level of *F. prausnitzii* showed lower expression of β-catenin, MMP-9 and NF-κB. Moreover, immunohistochemical analysis indicated that *KRAS* and *BRAF* expression were prominent in *F. nucleatum* and *B. fragilis* high abundance group, while *MLH1* showed lower expression. In conclusion, *F. nucleatum*, *B. fragilis* and *F. prausnitzii* can be identified as useful prognostic biomarkers for CRC, and dysbiosis might worsen the patients' prognosis by up-regulating gut inflammation level.

## INTRODUCTION

Colorectal cancer (CRC) is a common life threatening disease worldwide [1], with 700,000 annual mortalities making it the fourth most deadly cancer in both men and women [2]. In recent years, the 16S rRNA gene sequencing approach has been widely used as an effective tool to globally analyze the microbial community [3, 4], and multiple studies have demonstrated that breakdown of the intestinal microbiota structure can promote carcinogenesis and development of CRC [5–9]. Comparative data about microflora in relation to survival of patients with colorectal cancer are scanty, but may be of clinical significance. Flanagan et al. demonstrated a significant association between *Fusobacterium nucleatum* level and patient outcome and suggested that *F. nucleatum* may have value as a prognostic indicator [5]. Boleij et al. found that the detection of Bacteroides fragilis toxin (BFT), which was produced by *Enterotoxigenic Bacteroides fragilis* (ETBF), increased in the mucosa of later staged CRC [10]. These studies show there is a possibility that some type of microbe will affect the prognosis of patients with CRC. Given that infection has gradually been accepted as a major driver of inflammation, and various inflammatory mediators substantially contribute to metastasis [11, 12]. We hypothesize that inflammation might be the key point between microbiota and prognosis of CRC.

In this study, we examined the microbial structure in CRC clinical tumor samples and assessed the correlation of microbiota with clinicopathologic features and with patient survival. The status of *MLH1*, *BRAF* and *KRAS* expression, as well as inflammation related TNF-α, COX-2, MMP-9, β-catenin and NF-κB of cancer tissues were assessed to calculate their correlation with different microbial phylotypes. This approach may reveal the pathological process of how microbiota could affect the prognosis of CRC patients.

## RESULTS

### Diversity and structural changes of the tumor microbiota in CRC patients with different prognosis outcome

Libraries of 16S rRNA V4 region amplicon sequences from 180 CRC tumor samples were sequenced. A total of 16,854,578 high-quality and classifiable reads were obtained from this study, with an average of 93,636 reads per sample. At 3% dissimilarity level, a total of 41,628 OTUs in all samples and an average of 231 OTUs per sample were identified.

**Figure 1:**
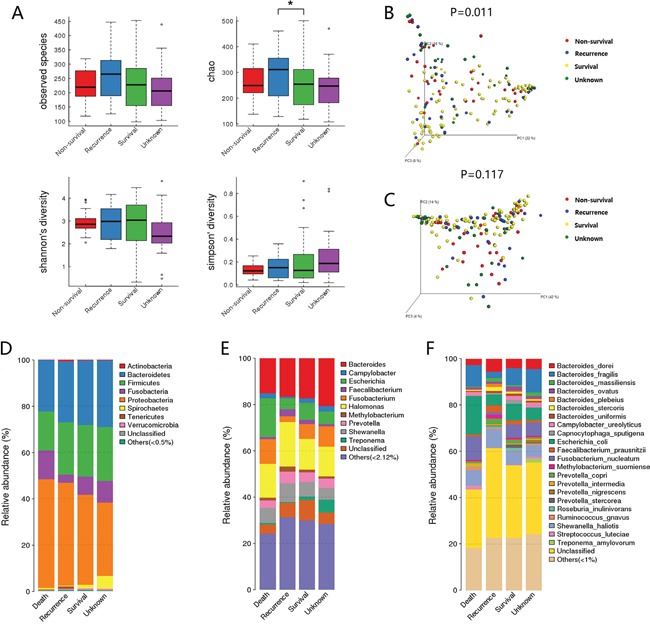
Diversity and structural changes of the tumor microbiota among the Non-survival group (n = 28), Recurrent group (n = 31), Survival group (n = 92) and Unknown group (n = 29) **A.** Alpha-diversity distances calculated using phylotype relative abundance measurements among groups. *: statistically significant *P* <0.05. Principal coordinates analysis (PCoA) scores plot of Bray-Curtis distance matrix **B.** and weighted Unifrac distance matrix **C.** based on the relative abundance of OTUs (97% similarity level). Each symbol represents a sample. Colors represent different groups. **D.** The dominant phyla of different groups. **E.** The dominant genera of different groups. **F.** The dominant species of different groups.

The value of Good's coverage for each group was over 99%. We examined the estimators of community richness (observed species and Chao indexes) and diversity and evenness (Shannon and Simpson indexes) among groups (Figure 1A). The only significant difference was detected between the survival group and recurrent group in Chao diversity index (Chao, 257 ± 88 vs. 397 ± 89, *P=* 0.03), demonstrating the significantly lower diversity found in survival group.

For beta diversity analysis, the microflora and compositions were analyzed and compared through the relative abundance of OTUs by using Bray-Curtis distance matrix and weighted Unifrac distance matrix for each group. Subsequent results of principal coordinates analysis (PCoA) exhibited the difference in bacterial community composition among groups. The first three principal component scores of Bray-Curtis distance matrix (Figure 1B) and weighted Unifrac distance matrix (Figure 1C) were 22%, 10%, 8% and 42%, 14%, 4%. Significant difference was detected in Bray-Curtis distance (*P*=0.011), suggesting that the community membership of each group was different.

The overall microbiota structure for each group at the phylum level is shown in Figure 1D. The dominant phyla of all groups were *Proteobacteria* (33.8%-49.4%), *Firmicutes* (16.9%-22.7%), *Bacteroidetes* (21.1%-27.9%), and *Fusobacterium* (3.38%-10.8%). When comparing the relative abundance of phyla among the groups, we found that the abundance of *Proteobacteria* was higher in survival group than in non-survival group (48.2% vs. 33.8%, FDR=0.063), while *Fusobacterium* was lower in survival group (3.38% vs. 9.71%, FDR=0.089), although the differences were not statistically significant.

The microbial composition was different at the genus level among groups. *Shewanella* (9.05% vs. 5.76%, FDR=0.091), *Methylobacterium* (2.54% vs. 1.56%, FDR=0.039), *Faecalibacterium* (2.99% vs. 0.93%, FDR=0.016) and *Sphingomonas* (1.38% vs. 0.79%, FDR=0.031) which constitute over 1% of the total bacteria in survival group, exhibited a relatively higher abundance than non-survival group. While *Fusobacterium* (9.23% vs. 2.70%, FDR=0.079) was relatively more abundant in non-survival group compared with survival group and the change was borderline significant. *Methylobacterium* (2.54% vs. 1.51%, FDR=0.09) and *Mycoplasma* (0.64% vs. 0%, FDR=0.01) showed higher abundance in survival group than in recurrence group. No other genus showed significant difference (Figure 1E).

In specie level, we found a higher level of *B. fragilis* (9.75% vs. 2.62%, FDR=0.017) in non-survival group than in survival group, while *F. prausnitzii* (2.96% vs. 0.92%, FDR=0.028) and *Methylobacterium suomiense* (1.91% vs. 0.78%, FDR=0.098) were more abundant in the survival group. Moreover, borderline statistic difference was found in *F. nucleatum* between non-survival group and survival group (5.66% vs. 1.08%, FDR=0.076) and *F. nucleatum* (5.10% vs. 1.08%, FDR=0.08) exhibited a greater abundance in the recurrence group than in survival group (Figure 1F).

### Correlation of microbiota in CRC patients with clinicopathologic features

The significant difference of microbiota between survival group and non-survival group showed that *B. fragilis* and *F. prausnitzii* might be correlated with patient's survival in CRC. Besides, *F. nucleatum*, a well-studied detrimental bacteria which could promote CRC development and progression, also showed higher abundance in non-survival group. Therefore, we evaluated the relationship between the level of the three bacteria and clinicopathologic characteristics of CRC patients. Based on the relative abundance of each microbiota in tumor sample, patients were divided into high and low bacteria subgroups with the median relative abundance as the cut-off, that was *B. fragilis* high vs *B. fragilis* low (Cut-off: 2.04%), *F. prausnitzii* high vs *F. prausnitzii* low (Cut-off: 0.55%), and *F. nucleatum* high vs. *F. nucleatum* low (Cut-off: 0.52%). As shown in Table 1, high abundance of *F. nucleatum* was significantly correlated with positive lymph node metastasis (*P* =0.011). Furthermore, high abundance of *F. prausnitzii* and *F. nucleatum* was significantly correlated with worse depth of invasion (*P* =0.015 and 0.015).

**Table 1:** Clinicopathological factors and microbiota in CRC patients

Characteristic	BH	BL	*P* value	FAH	FAL	*P* value	FUH	FUL	*P* value
Age			0.224			0.761			0.128
<60	32	40		35	37		31	41	
>=60	58	50		55	53		59	49	
Gender			0.543			0.761			0.361
Male	56	52		55	53		51	57	
Female	34	38		35	37		39	33	
Location			0.443			0.646			0.878
Colon	32	37		33	36		35	34	
Rectum	58	53		57	54		55	56	
Tumor size			0.168			0.443			0.092
<5cm	51	60		53	58		50	61	
>=5cm	39	30		37	32		40	29	
CEA			0.124			0.356			0.758
Normal	51	61		53	59		55	57	
Elevated	39	29		37	31		35	33	
Grade of differentiation			0.092			0.736			0.312
Well	61	71		65	67		63	69	
Poor	29	19		25	23		27	21	
Depth of invasion			0.083			0.015^*^			0.015^*^
T1 and T2	17	27		15	29		15	29	
T3 and T4	73	63		75	61		75	61	
Lymph node metastasis			0.036			0.901			0.011^*^
Negative	43	57		58	53		42	59	
Positive	47	33		42	37		48	31	
Remote Metastasis			1			0.278			0.718
Negative	86	86		84	88		85	87	
Positive	4	4		6	2		5	3	
TNM stage			0.052			0.296			0.101
I and II	41	54		44	51		42	53	
III and IV	49	36		46	39		48	37	

Bonferroni-correction: α=0.017

* statistically significant *P* <0.017

Abbreviation: BH: *B. fragilis* high, BL: *B. fragilis* low, FAH: *F. prausnitzii* high, FAL: *F. prausnitzii* low, FUH: *F. nucleatum* high, FUL: *F. nucleatum* low.

### Prognostic value of *B. fragilis, F. prausnitzii and F. nucleatum*

**Figure 2:**
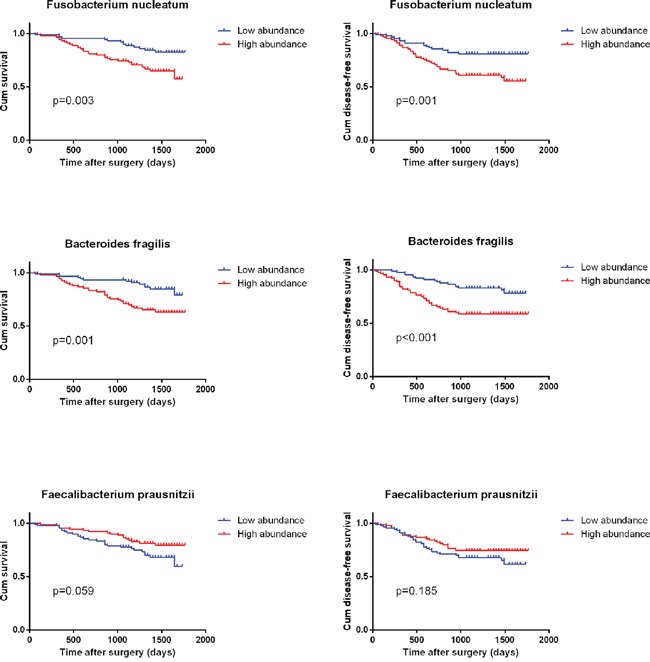
Kaplan–Meier survival curves for overall survival (OS) and disease free survival (DFS) in 180 CRC patients in relation to *B. fragilis, F. prausnitzii and F. nucleatum* level *P* values were obtained by log-rank test.

To assess the clinical significance of the three bacteria in CRC, Kaplan-Meier analysis and the log-rank test were used to analyze the relationship between bacteria relative abundance in cancer tissue and patient' survival. We found that the 3-year OS was significantly lower in patients with high *B. fragilis* and *F. nucleatum* than in those with low abundance of these two microbiota (*P=* 0.001, *P=* 0.003). And patients with low abundance of *F. prausnitzii* showed worse 3-year OS, although the difference was not significant (*P=* 0.06). Similarly, patients with high *B. fragilis* and *F. nucleatum* were significantly associated with poorer disease-free survival (DFS) rates than those with low abundance (P< 0.001, *P=* 0.001) (Figure 2). The univariate Cox regression analyses revealed that high *B. fragilis* (HR 2.888; *P=* 0.01), *F. nucleatum* (HR 2.533; *P=* 0.003), TNM stage (HR 2.325; *P=* 0.006) and carcino embryonie antigen (CEA) level (HR 1.945; *P=* 0.029) were associated with worse OS after radical surgery in these CRC patients. However, in the multivariate analysis, only *B. fragilis* (HR 2.010; 95% CI 1.020-3.961; *P=* 0.044), *F. nucleatum* (HR 1.993; 95% CI 1.024-3.879; *P=* 0.042) and TNM stage (HR 1.869; 95% CI 1.002-3.486; *P=* 0.049) was an independent predictor of the 3-year OS (Table 2). Furthermore, *B. fragilis*, *F. nucleatum* and TNM stage were associated with poor 3-year DFS both in univariate Cox regression analyses and multivariate analysis (Table 3).

**Table 2:** Univariate and multivariable Cox regression analyses for overall survival

Factor	Univariate analysis			Multivariate analysis		
	HR	95%Cl	*P*-value	HR	95%Cl	*P*-value
**Overall survival**						
Age(<60/>=60)	1.680	(0.879 to 3.213)	0.106			
Gender(male/female)	0.710	(0.376 to 1.339)	0,281			
Location(colon/rectum)	0.805	(0.431 to 1.503)	0.492			
Tumor size (<5cm/>=5cm)	1.588	(0.878 to 2.869)	0.129			
CEA(normal/elevated)	1.945	(1.076 to 3.515)	0.029*	1.757	(0.960 to 3.214)	0.067
Grade of differentiation (well/poor)	1.356	(0.719 to 2.558)	0.357			
TNM stage (I-II/III-IV)	2.325	(1.257 to 4.301)	0.006*	1.869	(1.002 to 3.486)	0.049*
*B. fragilis* (low/high)	2.888	(1.508 to 5.532)	0.001*	2.010	(1.020 to 3.961)	0.044*
*F. prausnitzii* (low/high)	0.562	(0.306 to 1.033)	0.059			
*F. nucleatum* (low/high)	2.533	(1.341 to 4.783)	0.003*	1.993	(1.024 to 3.879)	0.042*

HR relative risk, 95% CI 95% confidence interval.

* statistically significant P<0.05, Cox proportional hazard regression model.

**Table 3:** Univariate and multivariable Cox regression analyses for disease free survival

Factor	Univariate analysis			Multivariate analysis		
	HR	95%Cl	*P*-value	HR	95%Cl	*P*-value
**Disease-free survival**						
Age(<60/>=60)	1.421	(0.807 to 2.502)	0.216			
Gender(male/female)	0.736	(0.418 to 1.296)	0,281			
Location(colon/rectum)	0.880	(0.503 to 1.538)	0.651			
Tumor size(<5cm/>=5cm)	1.290	(0.752 to 2.213)	0.359			
CEA(normal/elevated)	1.495	(0.873 to 2.558)	0.147			
Grade of differentiation (well/poor)	1.678	(0.960 to 2.934)	0.078			
TNM stage (I-II/III-IV)	2.081	(1.203 to 3.599)	0.008*	1.823	(1.052 to 3.160)	0.032*
*B. fragilis* (low/high)	2.673	(1.504 to 4.751)	0.001*	2.042	(1.116 to 3.738)	0.021*
*F. prausnitzii* (low/high)	0.696	(0.406 to 1.194)	0.185			
*F. nucleatum* (low/high)	2.478	(1.395 to 4.404)	0.001*	1.829	(1.000 to 3.345)	0.050*

HR relative risk, 95% CI 95% confidence interval.

* statistically significant P<0.05, Cox proportional hazard regression model.

### Expression of inflammation related molecules in CRC tissues with different bacteria abundance

**Figure 3:**
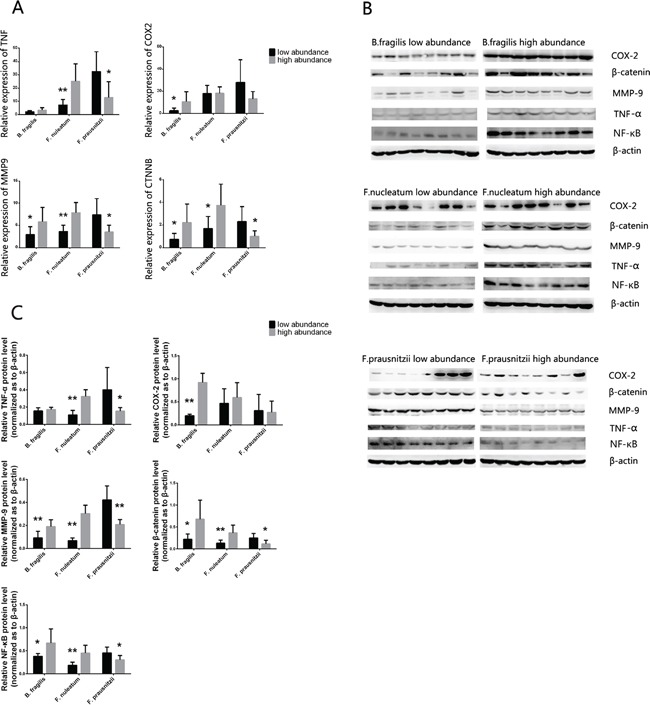
**A.** The mRNA expression level of the *TNF, COX2, MMP9* and *CTNNB* in cancer tissues with different abundance of *B. fragilis*, *F. nucleatum* and *F.prausnizii* were analyzed by RT-PCR, and the protein of TNF-α, COX-2, MMP-9, β-catenin and NF-κB were analyzed by western blot **B.** (*n* = 8 samples per group). Quantitation of the signals **C.** were analyzed based on the result of western blot. B-actin was used as the endogenous control. *P* values were calculated with Student's t test. *: statistically significant *P*<0.05, **: statistically significant *P*<0.01.

Quantitative RT-PCR was used to determine the expression of *TNF, COX2, MMP9* and *CTNNB* (catenin beta) in tumor samples. As shown in Figure 3A, *TNF* was overexpressed in CRC tissues of the *F. nuleatum* high abundance group (*P=*0.0024) and *F. prausnitzii* low abundance group (*P=*0.0117) compared to their opposite groups. Higher expression of *COX2* was found in *B. fragilis* high abundance group (*P=*0.0245) than in the low abundance one. And the expressions of *MMP9* and *CTNNB* were positively correlated with high abundance of *F. nuleatum* (*P=*0.0005 and *P=*0.0189) and *B. fragilis* (*P=*0.0432 and *P=*0.0300), but negatively correlated with high abundance of *F. prausnitzii* (*P=*0.0147 and *P=*0.0197). Consistently, western blotting analysis further confirmed the results obtained from qRT-PCR (Figure 3B). Moreover, the expression of NF-κB, which was detected by western blot, increased in *F. nuleatum* high abundance and *B. fragilis* high abundance groups, while decreased in *F. prausnitzii* high abundance group.

### Bacteria levels related to other molecular features of cancer tissue

**Figure 4:**
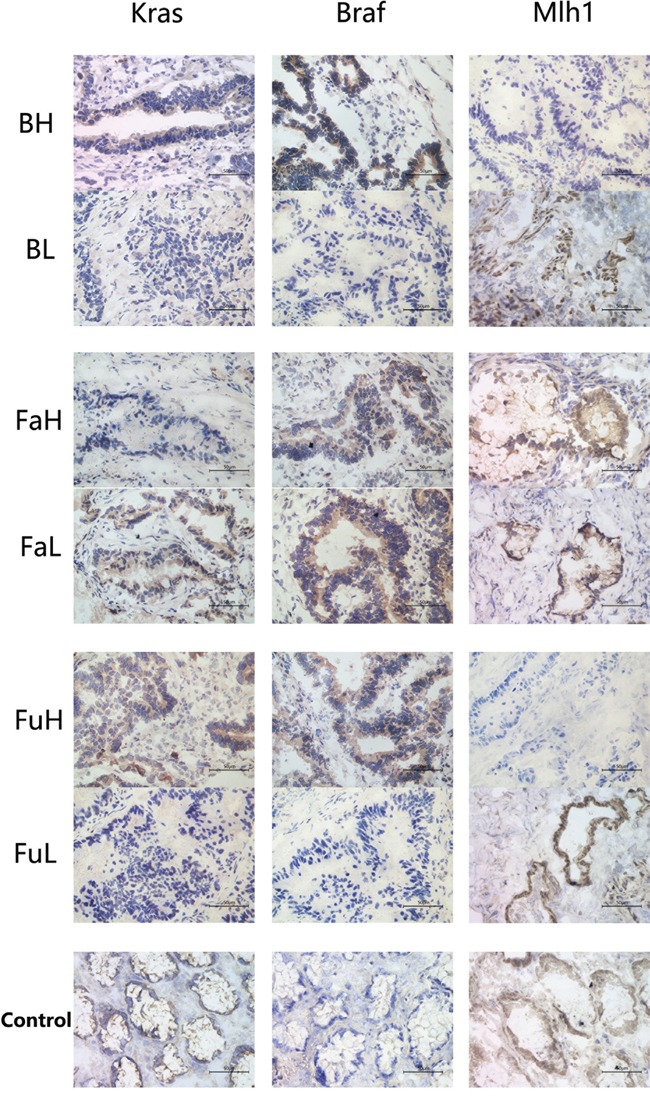
Immunohistochemistry was used to analysis the expression of *KRAS, BRAF* and *MLH1* in cancer tissues with different abundance of *B. fragilis, F. nucleatum* and *F.prausnizii* respectively And adjacent normal tissue samples were used as normal controls. Each group contains 6 samples, and each sample was made into 3 slides for staining. Abbreviation: BH: *B. fragilis* high, BL: *B. fragilis* low, FAH: *F. prausnitzii* high, FAL: *F. prausnitzii* low, FUH: *F. nucleatum* high, FUL: *F. nucleatum* low.

Immunohistochemistry was used to detect the differential expression of *KRAS*, *BRAF* and *MLH1* in cancer tissues (Figure 4). By comparing immunohistochemical scores of tumor samples with high or low bacteria abundance, we observed that *KRAS* was highly expressed in *F.nucleatm* high abundance group, *B. fragilis* high abundance group and *F. prausnitzii* low abundance group. The *F. nucleatm* high abundance group and *B. fragilis* high abundance group also exhibited a higher expression of *BRAF* and lower expression of *MLH1* (Table 4).

**Table 4:** Correlations of different bacteria abundance with IRS of *KRAS*, *BRAF* and *MLH1* expression

Group	Kras			Braf			Mlh1		
	Mean	SD	*P*-value	Mean	SD	*P*-value	Mean	SD	*P*-value
*B. fragilis* high abundance	4.333	0.0	0.0118*	6.667	0.857	0.0108*	1.667	0.615	0.0128*
*B. fragilis* low abundance	2.000	0.760		3.000	0.803		5.167	0.980	
*F. prausnitzii* high abundance	1.500	0.422	0.0025*	7.833	0.601	0.416	5.667	1.174	0.5036
*F. prausnitzii* low abundance	4.667	0.671		6.833	1.014		4.500	1.204	
*F. nucleatum* high abundance	3.667	0.422	0.0015*	5.500	0.806	0.0051*	2.333	0.803	0.0170*
*F. nucleatum* low abundance	1.333	0.333		1.333	0.843		6.000	1.000	

* statistically significant P<0.05

## DISCUSSION

Nowadays, cancer stage is the most important indicator for the prognosis of CRC patients, and multiple strategies were well designed based on different TNM stage. However, new tool to indicate more accurate clinical characteristics and effective therapeutic targets are still needed, so it is meaningful to find new predictor of CRC patients' prognosis which might lead to novel treatment method to improve patients' survival. In our study, we are the first to compare the microbial population among groups of cancer tissues divided by different post-operation prognosis status. Differences of *F. nucleatum*, *B. fragilis* and *F. prausnitzii*, found between non-survival and survival group, drew our special attention, because these three bacteria have relative high abundant, all of which counted for more than 1% of microbiota in species, and they are intimately related with colorectal cancer [5-8, 13, 14]. In addition, we found a correlation between high abundance of *F. nucleatum* and increased lymph nodes metastasis rates, which was consistent with previous study [15]. Tumor's depth of invasion was shown to be correlated with high *F. nucleatum* and low *F. prausnitzii* abundance. Further survival analysis confirmed the prognostic value of *F. nucleatum*, *Fragilis* and *F. prausnitzii*.

Another study has shown that patients with high levels of *F. nucleatum* had a significantly shorter survival time, which was similarly with our result [5]. But the number of patients enrolled in the cohort of that study was relatively small (32 patients). A more recent study reported a similar result using a larger databases of CRC cases in USA, which observed a correlation between high amount of tissue *F. nucleatum* DNA and higher CRC-specific mortality [16]. This result was promising, but the positive rate of bacteria DNA was relatively small which made the positive group less representative. ETBF, a major subtype of *B. fragilis*, was also associated with CRC through producing BFT [17]. A recent study suggested that *BFT* gene positivity was more prominent in later stage CRC, which showed a possible link between increased *B. fragilis* and worse prognosis of CRC [10]. In our study, we report, for the first time, that the high abundant of *B. fragilis* is correlated with poor patient's clinical outcome. Furthermore, increased *F.prausnitzii*, a well-known human intestinal probiotic bacteria, was shown to be related with better survival status, a result that has not been reported before.

The specific mechanisms by which gut microbiota affects the development of CRC are still not well understood. One of the most promising theories is that it is thought to be through microbe-driven intestinal inflammation [18]. Interestingly, *F. nucleatum*, *B. fragilis* and *F. prausnitzii* are all key players in modifying intestinal inflammation levels [6, 7, 19]. In a study about colorectal adenomas, the abundance of *F. nucleatum* was found to positively correlate with inflammatory cytokine genes expression including that of *TNF*, which was consistent with our result [20]. TNF-α is produced during the inflammatory response and can promote survival, attachment, and proliferation of metastatic colon cancer cells in a mouse model of lung metastasis depending on the activation of NF-κB by inflammation and cancer cells [21]. Moreover, through activation of NF-κB and STAT3, TNF-α can enhance epithelial-mesenchymal transition which are critical steps that allow polarized epithelial tumor cells to become mesenchymal like, enhancing cell migration and invasion [22, 23]. Meanwhile, MMP-9 was also elevated in *F. nucleatum* high abundant group. MMP-9, known as an independent stimulus for increased cell migration [24], can be activated by inflammatory signals, such as NF-κB [25]. And, as previously mentioned, NF-κB can be activated by TNF-α in certain tumor microenvironment [21]. Furthermore, it has been demonstrated that *F. nucleatum* can bind to E-cadherin on epithelial cells via FadA and activates β-catenin which is a transcription factor in the Wnt signal transduction pathway [6], Wnt signaling is fundamental in CRC progression [26]. In addition, studies suggest that *F. nucleatum* can also generate a proinflammatory microenvironment by recruit tumor-infiltrating immune cell [27] and downregulate antitumor T cell-mediated adaptive immunity [28] to promote CRC progression.

*B. fragilis*, was also related with higher *CTNNB* expression in our study. BFT can alter epithelia structure and function including cleavage of the tumor suppressor protein, E-cadherin, resulting in enhanced nuclear Wnt/β–catenin signaling that yields increased colonic carcinoma cell proliferation and metastasis [29–31]. PGE2, a major downstream mediator of COX-2, can activates β-catenin-dependent signaling, which promotes survival and proliferation. Besides increased COX-2 can also stimulate tumor angiogenesis by inducing production of VEGF and basic fibroblast growth factor, and it can increase tumor dissemination by altering the adhesive properties of cells and increasing matrix metalloproteinase activity [32, 33]. Available evidence demonstrated that BFT, through activation of NF-κB, could stimulate intestinal epithelial cells to induce the expression of COX-2 and increased release of PGE2 [13]. In the meantime, MMP-9 is also elevated in the *B. fragilis* high abundance group which might also be explained by activation of NF-κB that was induced by BFT.

Several studies have shown that culture supernatant of *F. prausnitzii* exerts an anti-inflammatory effect both *in vitro* and *in vivo* [34]. A recent experiment demonstrated that a 15 kDa protein produced by *F. prausnitzii* possessed anti-inflammation properties through inhibition of NF-κB pathway in intestinal epithelial cells in an animal model [35], which is consistent with our finding. Notably, the down regulated NF-κB could decrease the expression of various inflammation related factors including β–catenin and MMP-9 [25, 36], both of which showed lower expression level in *F. prausnitzii* high group in our experiment and could promote CRC metastasis. Furthermore, *TNF* was also overexpressed in *F. prausnitzii* low group, which further supports the idea that *F. prausnitzii* possess an anti-inflammation effect, although the potential mechanism is not thoroughly understood.

Evidences have shown that overexpression of *KRAS* and *BRAF* were markers of poor prognosis [37, 38] which were fortunately consistent with our findings of prognostic values of *F. nucleatum*, *B. fragilis* and *F.prausnitzii*. In addition, MSI, the primary causes of which is hypermethylation of the *MLH1* promoter, was also associated with clinical outcomes of CRC [39, 40], and in this study, decreased expression of Mlh-1 had also been detected in *F. nucleatum* high and *B. fragilis* high abundant samples. These findings might imply that cancers which are accompanied with high abundance of *F. nucleatum* and *B. fragilis* or low abundance of *F. prausnitzii* are more invasive and inclined to metastasis. Furthermore, serrated adenocarcinoma, a subtype of CRC which is developed though serrated pathway, is characterized by high frequency of *KRAS* and *BRAF* mutation and *MLH1* deficiency [41, 42]. Evidence showed that this kind of CRC is likely to have less favorable 5-year survival [43]. Therefore, it came a question that whether there's an association between bacteria *F. nucleatum* or *B. fragilis* and serrated adenocarcinoma, which might be worth exploring in future studies.

Moreover, as obvious benefits of anti-epidermal growth factor receptor therapy were shown in patients with *KRAS* mutations [44, 45] and sensitivity to irinotecan was found in the *MLH1* deficiency cell model [46, 47], our finding poses the question as to whether patients that suffered from dysbiosis should receive more aggressive chemotherapy? This might be a promising direction of future microbiotic study.

The study has some limitations. First, our findings based on patients in a single center, and the prognostic value of these bacteria might require a multi-center study with larger data to validate this result. Second, the specific mechanism by which bacteria promote invasion and metastasis of CRC and unknown mutual effect that exist among different bacteria will surly need to be explored by well-designed animal and cell line experiments in the future.

In conclusion, our study is the first report demonstrating the prognosis value of *B. fragilis* and *F.prausnitzii*, and further validates the connection of *F. nucleatum* and cancer-specific mortality. Furthermore, the correlation between bacteria's relative abundance and inflammatory factors suggests that microbiota might impact patient's prognosis via inducing gut inflammation. Moreover, given that the tumor samples could be easily collected both in surgery and colonoscopy, our results will be useful in developing novel bacteria-related prognostic indicator for CRC, and encourage further investigation of the role played by microbiota in CRC pathology.

## MATERIALS AND METHODS

### CRC patients

A total of 180 CRC patients were enrolled in our study. Patients with stages I–III cancer subjected to standard curative surgery, while stage IV CRC tissues were collected from patients who received palliative surgery to relieve serious cancer related contradiction. Surgeries were performed at the general surgery department of Affiliated Hospital of Qingdao University between 2010 and 2012, and all patients who received postoperative treatment were guided by the National Comprehensive Cancer Network Guidelines. Of all the patients, there were 108 males and 72 females with a mean age of 62.2 (age range 30–88 years). The median follow-up period was 47 months with a range from 36 to 59 months. The criteria for study enrollment were histopathological diagnosis of primary CRC, newly diagnosed and untreated, no history of other tumors, and the potential to follow up. Patients who used antibiotics within 2 months before operation, or were regularly using Non-steroidal anti-inflammatory drugs, statins or probiotics were excluded from the study. Other exclusions included chronic bowel disease, other signs of infections, food allergies and dietary restrictions.

### Sample preparation

CRC tumor samples and adjacent normal tissue samples (at least 5cm from the tumor site) of these 180 patients were obtained from the gastrointestinal cancer specimen bank of Affiliated Hospital of Qingdao University, Qingdao, China. To be specific, surgically resected specimens were collected immediately after tumor removal and stored at −80°C until use. The TNM staging were determined according to the American Joint Committee on Cancer system and all specimens were graded histologically according to the World Health Organization classification criteria. Written informed consents of joining the specimen bank were obtained from all the patients before surgery, and the protocols used in the study were approved by the Ethics Committee of Affiliated Hospital of Qingdao University. Clinical and pathologic data were reviewed from gastrointestinal cancer database of Affiliated Hospital of Qingdao University, Qingdao, China.

So as to preliminarily detect the species of bacteria with prognostic value, subjects included in the study were subdivided based on their different survival conditions. From those, 92 patients corresponded to the survival group (people who lived more than 3 years without any sign of recurrence or metastasis), 28 to the non-survival group (people who died within 3years after surgery for CRC related causes), 31 to the recurrence group (people who experienced recurrence or metastasis of primary tumor within 3 years but survived), and 29 to the unclear group (people who lived more than 3 years with unclear history of recurrence or metastasis).

### DNA extraction

DNA was extracted from all tumor samples using CTAB method with minimal modification. Concentration of DNA was measured by fluorometer or microplate reader, and sample integrity was tested by agarose gel electrophoresis (1% concentration of agarose Gel: 1 %; 150 V; 40 min electrophoresis time). All DNA samples were stored at −20°C until used.

### PCR and sequencing analysis

Amplification of the V4 region of the bacterial 16S rRNA gene was performed by polymerase chain reaction (PCR) using universal primers 319F and 806R. The reaction mix consisted of Phusion High-Fidelity PCR Master Mix (NEB, Ipswich, MA, USA) and appropriate primer/probe pairs. The PCR program was as follows: 3 min denaturation at 98°C followed by 30 cycles of 45 s at 95°C (denaturation), 45 s for annealing at 55°C and 45 s at 72°C (extension), with a final extension at 72°C for 7 min. The PCR products were purified with AMPure XP beads (Agencourt Bioscience) to remove the unspecific products prior to library construction. The library was quantitated in two ways: the average molecule length was determined using the Agilent 2100 bioanalyzer instrument (Agilent DNA 1000 Reagents), and then quantified by real-time quantitative PCR (qPCR; EvaGreen TM). Sequencing of qualified libraries was performed by the BGI-Huada Genomices institute in Shenzhen using MiSeq System, with the sequencing strategy PE250 (PE251+8+8+251) or PE300 (PE301+8+8+301) (MiSeq Reagent Kit).

### Bioinformatics analysis

The sequences were clustered into operational taxonomic units (OTU) with a 97% threshold by using USEARCH (v7.0.1090) [48], and the OTU unique representative sequences were obtained. Chimeras were filtered out by using UCHIME (v4.2.40). [49] Representative OTUs were aligned to the optimized sequences and the abundance of OTUs per samples was obtained for performing further analysis. Ribosomal Database Project (RDP) Classifier v.2.2 was used to taxonomically classify OTU representative sequences in the following databases: Greengene V201305; [50] RDP (Release9 201203) [51].

### Quantitative real-time PCR

In order to compare different expression level of *TNF, COX2, MMP9*, and *CTNNB* in 180 cancer tissues with different abundance of bacteria associated with patients' prognosis, we defined 30 samples with most abundance of certain bacteria species as high abundance, similarly 30 samples with lest abundance of same species were defined as low abundance. Then, eight tissue samples were randomly chosen from each of the two sets of samples to build two group of specimens that contained high and low bacterial species abundance. Quantitative real-time PCR was used to detect the different mRNA expression level of *TNF*, *COX2*, *MMP9*, and *CTNNB* between these two groups. Briefly, total RNA was extracted using TRIzol reagent (Invitrogen). The reverse-transcription PCR (RT-PCR) was performed using Fermentas RT reagent Kit according to the manufacturer's instructions, and the qPCR was performed using the SYBR Green 1 and measured using LightCycler 480 System (Roche, Basel, Switzerland). Primers for qRT-PCR are as follows. *TNF*: Forward, 5′-GCCGCATCGCCGTCTCCTAC-3′, Reverse 5′-CCTCAGCCCCCTCTGGGG TC-3′, *MMP9*: Forward, 5′- TTGACAGCGACAAGAAGT-3′, Reverse 5′- GGGCGAGGACCATAGA-3′, *COX2*: Forward, 5′- TACAATGCTGACTATGGCTAC-3′, Reverse 5′- TGATGCGTGAAGTGCTG-3′, *CTNNB*: Forward, 5′- GGCAGCAACAGTCTTA -3′, Reverse 5′- GTCTCAGGGAACATAGC -3′, *Actin*: Forward, 5′- GGAAATCGTGCGTGACATTAA -3′, Reverse, 5′- AGGAAGGAAGGCTGGAAGAG -3′. The relative expression levels were calculated by the 2^−ΔΔCT^ method. Each assay was carried out in triplicate.

### Western blot analysis

Western blotting was performed to detect the differences of TNF-α, COX-2, MMP-9, β-catenin and NF-κB in above-mentioned paired tissues. Total proteins were prepared from frozen tissue by Cellytic M cell lysis Reagent (Sigma-Aldrich Inc., St. Louis, MO, US). After being centrifuged at 12,000g for 20 min, the supernatants were loaded onto 10% SDS-PAGE gels, electrophoresed, and transferred to PVDF membrane (Millipore). Then, Membranes were incubated overnight at 4°C with TNF-α, COX-2, MMP-9, β-catenin and NF-κB antibodies (Abcam) respectively, β-actin was served as an internal loading control. After washes, membranes were incubated with appropriate secondary antibodies for 1 hr at room temperature, and bands were scanned using a ChemiDoc™ Touch Imaging System (Bio-Rad Laboratories, UK).

### Immunohistochemical analysis

Briefly, tumor samples were embedded using tissue freezing medium, then fixed tissues were cut into sections by a microtome. The endogenous peroxidase activity was blocked with 3% H_2_O_2_ for 20 min and pre-incubated in normal goat serum for 20 minutes at room temperature. After blocking, the sections were incubated with primary anti-Kras (Millipore), anti-Braf (Abcam) and anti-Mlh1 (Abgent) antibody at 4°C overnight. After rinsing three times with 0.01 mol/L phosphate-buffered saline (PBS; pH = 7.4) for 10 mins, the detection of the primary antibody was achieved by addition of biotinylated secondary antibody for 1 hr at room temperature, and stained with DAB (3,3-diaminobenzidine) after washing in PBS again. The staining results were scored by two pathologists blinded to the clinical data using the German immunoreactive score (IRS). Briefly, staining intensity was graded as “0” (negative), “1” (weak), “2” (moderate) and “3” (strong); staining extent was graded as “0” (<5%), “1” (5–25%), “2” (25–50%), “3” (50–75%) or “4” (>75%). Values of the staining intensity and the staining extent were multiplied as a final IRS.

### Statistical analysis

Metastats (http://metastats.cbcb.umd.edu/) and R (v3.0.3) are used to determine which taxonomic groups were significantly different between groups of samples. We adjusted the obtained *P*-value by a Benjamini-Hochberg false discovery rate (FDR) correction (function '*P*.adjust' in the stats package of R (v3.0.3)) [52]. Continuous data are presented as mean±standard deviation, unless otherwise stated. The *P*-values for Bray-Curtis distance and Weighted-Unifrac distance were calculated by ANOSIM analysis. Differences between groups were analyzed using Student t test (two-tailed). The association between clinicopathological variables and differences in microbiota were examined by χ 2 tests. The categorical data were analyzed by a Fisher's exact test. Overall survival (OS) curves were analyzed using the Kaplan–Meier method, and differences were examined using log-rank tests. Cox's proportional hazard regression test was used to estimate univariate and multivariate hazard ratios for prognosis. *P* values were two sided, and those <0.05 were considered statistically significant. Analyses were performed with the SPSS software 17.0 and GraphPad Prism 5 software package (GraphPad software Inc, San Diego, CA, USA).
